# In vitro evaluation of the effectiveness of Schinus molle essential oil against Candida albicans: resistant and sensitive strains

**DOI:** 10.21142/2523-2754-1303-2025-248

**Published:** 2025-08-31

**Authors:** Carlos Alejandro Cadena Viteri, Katherine de los Ángeles Cuenca León, Edisson Mauricio Pacheco Quito, Miriam Verónica Lima Illescas

**Affiliations:** 1 Especialización en Ortodoncia, Universidad Católica de Cuenca. Campus Universitario Azogues, Ecuador. carlos.cadena.47@est.ucacue.edu.ec Universidad Católica de Cuenca Especialización en Ortodoncia Universidad Católica de Cuenca Ecuador carlos.cadena.47@est.ucacue.edu.ec; 2 Unidad Académica de Salud y Bienestar, Facultad de Odontología, Universidad Católica de Cuenca. Cuenca, Ecuador. kcuencal@ucacue.edu.ec, epachecoq@ucacue.edu.ec, mlimai@ucacue.edu.ec Universidad Católica de Cuenca Unidad Académica de Salud y Bienestar Facultad de Odontología Universidad Católica de Cuenca Cuenca Ecuador kcuencal@ucacue.edu.ec epachecoq@ucacue.edu.ec mlimai@ucacue.edu.ec

**Keywords:** Anti-Bacterial Agents, Candida albicans, Orthodontics, Phytotherapy, agentes antibacterianos, Candida albicans, ortodoncia, fitoterapia

## Abstract

**Objective:**

The purpose of this research project was to establish the inhibitory effect of the essential oil and ethanolic extract of *Schinus molle* against *Candida albicans* resistant strain (ATCC® 60193™) and *Candida albicans* sensitive strain (ATCC® 90028™).

**Materials and methods:**

An in-vitro experimental study was carried out to assess the antimicrobial activity of the formulations of essential oil and ethanolic extract of *S. molle* at concentrations of 25%, 50%, 75% and 100%, against resistant and sensitive *Candida albicans*. 70% alcohol (negative control) and fluconazole (positive control) were used as controls. Regarding the statistical analysis, measures of central tendency and dispersion were assessed. For the relationship between the type of extract, species of *Candida albicans* and concentration, the Kruskal-Wallis test was used, with a confidence level of 95% (p < 0.05).

**Results:**

A clinical inhibitory effect was observed at various concentrations for both the oily and ethanolic extracts of Schinus molle. However, statistical analysis indicated that these effects were not significant, except at 100% concentration. The 100% ethanolic extract of *S. molle* exhibited the highest mean inhibition value against resistant Candida albicans (5.67 ± 4.509), with a statistically significant difference (p < 0.05, p = 0.038). Nevertheless, its inhibitory effect did not surpass that of fluconazole.

**Conclusions:**

The 100% ethanolic extract of *S. molle* presented a higher inhibitory effect than the other concentrations against resistant *Candida albicans*. However, its efficacy did not exceed that of fluconazole, which acted as a positive control. Additional studies are recommended to further explore the potential therapeutic benefits and expand the understanding of its antimicrobial potential.

## RESUMEN

**Objetivo:**

El propósito de este proyecto de investigación fue establecer el efecto inhibitorio del aceite esencial y el extracto etanólico de *Schinus molle* contra *Candida albicans* cepa resistente (ATCC® 60193™) y *Candida albicans* cepa sensible (ATCC® 90028™).

**Materiales y métodos:**

Se realizó un estudio experimental *in vitro* para evaluar la actividad antimicrobiana de las formulaciones de aceite esencial y extracto etanólico de *S. molle* a concentraciones del 25%, 50%, 75% y 100%, contra *Candida albicans* resistente y sensible. Se utilizaron como controles alcohol al 70% (control negativo) y fluconazol (control positivo). En cuanto al análisis estadístico, se evaluaron medidas de tendencia central y dispersión. Para la relación entre el tipo de extracto, especie de *Candida albicans* y concentración, se utilizó la prueba de Kruskal-Wallis, con un nivel de confianza del 95% (p < 0,05).

**Resultados:**

Se observó un efecto inhibitorio clínico a diversas concentraciones, tanto para los extractos oleoso como etanólico de *Schinus molle*. Sin embargo, el análisis estadístico indicó que estos efectos no fueron significativos, excepto al 100% de concentración. El extracto etanólico al 100% de *S. molle* presentó el mayor valor medio de inhibición frente a *Candida albicans* resistente (5,67 ± 4,509), con una diferencia estadísticamente significativa (p < 0,05, p = 0,038). No obstante, su efecto inhibitorio no superó al del fluconazol.

**Conclusiones:**

El extracto etanólico al 100% de *S. molle* presentó un mayor efecto inhibitorio que las otras concentraciones frente a *Candida albicans* resistente. Sin embargo, su eficacia no superó la del fluconazol, que actuó como control positivo. Se recomiendan estudios adicionales para explorar con mayor profundidad los posibles beneficios terapéuticos y ampliar la comprensión de su potencial antimicrobiano.

## INTRODUCTION

In dentistry, one of the most frequent pathologies are carious lesions followed by periodontal disease, especially in patients with poor plaque control. White spot lesions have been identified for several years as a common and undesirable morbidity in patients with fixed orthodontic appliances. Its etiology is directly related to the characteristics of the intraoral system, which acts as a physical means of retaining food and bacterial plaque in structures such as archwires, bands and brackets. This condition is aggravated by inadequate oral hygiene, which can lead to the progression of the dental clinical picture and increase the risk of complications. ^1^^-^^3^

On the other hand, there are pharmacological therapeutic options for conventional use with antifungal effectiveness against *Candida albicans* at the oral level. One of the main disadvantages of these synthetic drugs is the presence of systemic side effects, the most frequent being hepatobiliary and gastrointestinal disorders. ^4^^-^^6^

Phytotherapy has gained a prominent place in the field of health due to the use of active chemical compounds present in plants, which have shown significant effects on various pathogenic microorganisms. Among these, essential oils have shown great potential as adjuvants in the treatment of oral conditions, including white spot lesions, gingivitis and periodontal disease. ^7^^-^^12^

Oily extracts are considered volatile metabolites with the particularity that they usually have aroma and flavor. The active principles present in oily extracts are formed in certain cytoplasmic organelles as well as in the cell plastids of plant species. One of the main active mechanisms of these oily extracts is the detriment in the cellular membrane of microorganisms thanks to the excellent facility to cross the two phospholipid layers of the cellular membrane. There are reports that oily extracts that work against fungi affect the amount of ergosterol in the fungal cell membrane. Similarly, terpenoids hinder enzymatic reactions to produce energy for the microorganism. ^4^^,^^9^^,^^13^^-^^16^

Schinus molle, a plant species also known as false pepper, pink pepper or Peruvian pepper, is native to the subtropics, especially in South America, and belongs to the Anacardiaceae family. According to reports in scientific literature, this plant possesses several beneficial pharmacodynamic properties, including antioxidant, anti-inflammatory, antifungal and antibacterial activities. In addition, its essential oil and ethanolic extract have been reported to act as nervous system stimulants. It is also attributed to the ability to stimulate fibroblast activity, promoting rapid and effective healing. ^7^^,^^15^^,^^17^^-^^24^

The ethanolic extracts of this plant species act through the phenolic acids that this plant possesses, which are very effective against bacterial species, especially gram-positive, thanks to the position and type of substituent of the benzene ring, in addition, they can suppress the growth of microorganisms since they have pro-oxidative characteristics, thus inhibiting bacterial growth and causing cell lysis. ^19^^,^^25^^-^^28^

In this context, the oily extract of *Schinus molle* is attracting increasing interest thanks to its multiple therapeutic properties, which underlines the importance of its study in the development of natural and effective alternatives for oral health care. ^7^^-^^12^

In recent years phytotherapy has made great progress in the field of stomatology, combating bacterial, parasitic and fungal infections. White spot lesions that may develop due to the increase of cariogenic bacteria, generally accompanied by fungi during orthodontic treatment, can be reduced with herbal mouthwashes, however, it should go hand in hand with the mechanical removal of bacterial plaque to prevent the development of these bacteria and opportunistic fungi such as *Candida albicans*. ^12^^,^^19^^,^^29^^-^^33^

*Candida albicans*, as an integral part of the oral microbiota, is categorized as an opportunistic fungus when dysbiosis occurs, resulting in a significant increase in its colonization. This phenomenon is particularly common in patients under antibiotic treatment and is aggravated in those with immuno-compromised systems. ^5^^,^^13^^,^^31^

This microorganism shows a marked affinity for patients with acrylic prostheses and has been identified as a contributing factor in the development of dental caries lesions. The scientific literature reports a frequent coexistence between *Streptococcus mutans* and *Candida albicans*, particularly in patients who use metallic orthodontic appliances. These orthodontic devices contribute to significant alterations in the balance of the oral microbiome, favoring an environment conducive to colonization and activity of both microorganisms. ^4^^,^^6^^,^^8^^,^^29^^,^^30^

Although the use of antibiotics and antifungals is essential in certain pathologies that affect the patient locally or systemically, their prescription must be carried out under strict criteria. However, in our environment, access to these drugs is free and lacks adequate control by the competent regulatory bodies. This situation increases the risk of developing both bacterial and fungal resistance, which represents an imminent threat to public health in the medium term. ^4^^,^^21^^,^^31^^,^^34^

Phytotherapy in recent years has been gaining more relevance, however, at present fluconazole is still considered as the first line drug against this opportunistic fungus, which is why it has been decided to search for essential extracts and ethanolic plant extracts for the development of mouthwashes that can help reduce the fungal load without having a great alteration of the oral bacterial flora. ^8^^,^^31^^,^^32^^,^^34^

In previous studies, *Candida albicans* strains were genetically manipulated, resulting in “gain of function”, i.e., they were molecularly modified to acquire azole resistance, thus demonstrating that essential oils, such as cinnamon, were more effective than fluconazole in certain cases of fungal resistance by *C. albicans*. ^7^^,^^31^^,^^35^^-^^38^

The present research is based on an existing gap in scientific knowledge regarding the potential inhibitory effect of the essential oil and ethanolic extract of this plant on resistant *Candida albicans*. This strain, considered an opportunistic fungus of high clinical relevance, could be present in patients undergoing orthodontic treatments in the postgraduate clinic of the Catholic University of Cuenca, Azogues campus. In this context, the main objective is to evaluate this antifungal activity, providing evidence that could have relevant therapeutic implications.

## MATERIALS AND METHODS

This is a longitudinal laboratory study carried out in the laboratories of the Center for Research, Innovation and Technology Transfer (CIITT) of the Catholic University of Cuenca. This research project was considered exempt from requiring approval by the bioethics committee. We worked with oily extract (OE) obtained from the U.S. company dōTERRA. They are extractedunder a quality protocol (CPTG) “Certified and Tested as Pure and Quality” to ensure the purity and quality of each batch, thus including multiple phases of testing to ensure that the oils are free of contaminants, synthetic fillers and other unwanted components.

The ethanolic extract (e) was obtained from the Bacterial and Microbiology In-Med (BMI) laboratory in Quito, Ecuador. 

**Figure 1:**
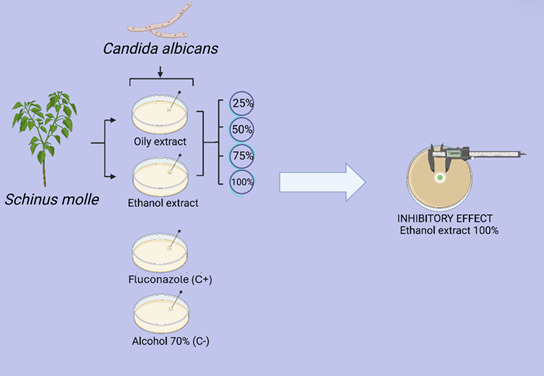
Experimental scheme

Both the oily and ethanolic extracts were stored in a cold environment away from light to avoid decomposition and maintain stability. These extracts were diluted, except for 100%, with 70% alcohol to obtain concentrations of 25%, 50%, 75%. (v/v), as shown in Figure 1.

Two species of microorganisms were used, both of synthetic origin, resistant *Candida albicans* (ATCC®60193) (CR) and sensitive *Candida albicans* (ATCC®90028) (C). Fluconazole was used as positive control, and 70% alcohol was used as a negative control.

It was performed in triplicate for oily extract of *S. molle* against resistant *Candida albicans*, ethanolic extract of *S. molle* against resistant *C. albicans*; in duplicate for oily extract against sensitive *Candida albicans*, in each of the following concentrations. 

To ensure biosafety and complete inactivation of biological agents in order to eliminate, mitigate and reduce the associated risks and guarantee the health of the population, the petri dishes were subjected to an autoclave sterilization process at 121 °C, with a pressure of 15 psi, for 30 minutes. This procedure was performed under strict biosafety measures.

### Inclusion criteria

Microorganisms of synthetic origin both resistant *Candida albicans* (ATCC®60193) and sensitive *Candida albicans* (ATCC®90028), pure oily and ethanolic extract (*Schinus molle*).

### Exclusion criteria 

Oil extract and ethanolic extract of other plant species. Clinical isolation of *Candida albicans* of human origin, and samples contaminated with other types of microorganisms.

## MICROBIOLOGICAL ANALYSIS

The mechanism by which *C. albicans* becomes resistant is through alterations in target enzymes through a mutation in the ERG11 gene, which encodes lanosterol 14α-demethylase, thus producing a reduction in the affinity of the azoles for their site of action, thereby decreasing the efficacy of the treatment. Also, the overexpression of efflux pumps, through the increase in the expression of genes such as CDR1, CDR2 and MDR1, which are encoding genes for these pumps, thus actively expelling the intracellular antifungals, reducing their concentration and therefore their effectiveness. It has also been identified that they can develop alterations in the ergosterol biosynthesis pathway allowing the survival of the fungus despite conventional treatment, especially with fluconazole. ^3^^,^^39^

**Figure 2:**
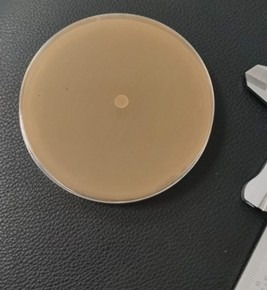
Alcohol 70% (negative control)

**Figure 3:**
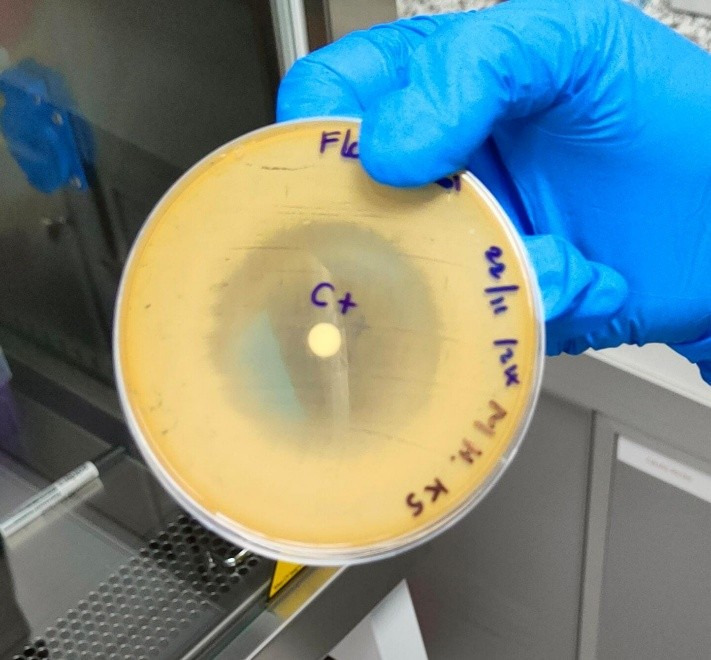
Fluconazole (positive control)

Two strains were used, one resistant *Candida albicans* (ATCC® 60193™) (CR) and one sensitive *Candida albicans* (ATCC® 90028) (C). The specific agar for this species was prepared following the manufacturer's instructions. The dilution made from the ethanolic extract (e) and oily extract (AE) of *Schinus molle* was 25%, 50%, 75%; that of 100% was not diluted. Alcohol at 70% was used as solvent, in the different concentrations mentioned above. Fluconazole in this case was a positive control as shown in Figure 3 and 70% alcohol was used as a negative control as shown in Figure 2.

The strains of the microorganisms of synthetic origin were reactivated and then replicated in the monopetri boxes and inoculated in the blank antibiogram discs at the different concentrations already mentioned. It was carried out in triplicate for oily extract of *S. molle* against resistant *Candida albicans*, ethanolic extract of *S. molle* against resistant *C. albicans*; in duplicate for oily extract against sensitive *Candida albicans*, in each of the concentrations. 

The monopetri boxes were placed in an oven for approximately 72h at 24^o^C, after which the presence of inhibitory halos was observed. The halos were measured using a vernier calibrator.

### Data collection and statistical analysis

For the statistical analysis, measures of central tendency and dispersion were evaluated using SPSS, v25. For the relationship between the type of extract, *Candida albicans* species and concentration, the Kruskal-Wallis test was used, with a confidence level of 95% (p < 0.05). Microsoft Excel was used for the edition of tables and figures.

## RESULTS

The present investigation aimed to observe the inhibitory effectiveness of the oily extract (OE) and ethanolic extract (e) of *S. molle* against resistant *Candida albicans* (RC) and sensitive *Candida albicans* (C). 

Descriptive statistical data of the inhibitory halo (mm) for the different solutions evaluated are shown. The value of the halos obtained for the positive control was 19 mm while for the negative control it was 0 mm. 

**Figure 4:**
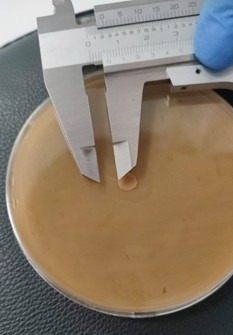
100% ethanolic extract against resistant *C. albicans*. Halo 6mm.

**Figure 5:**
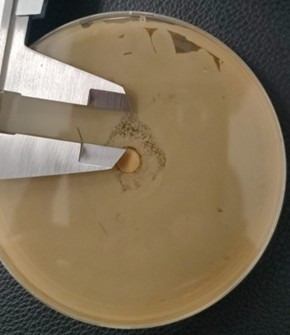
100% ethanolic extract against resistant *C. albicans*. Halo 10mm

Similarly, the following results were obtained at a 100% concentration of both the oily extract and the ethanolic extract against RC and C, as shown in Figure 4 and Figure 5. The highest mean value was that of ethanolic extract of *Schinus molle* against resistant *Candida albicans* (5.67 ± 4.509), giving us a value of *p<0.05 (*p=0.038) thus showing us that there was statistically significant dissimilarity as Table 1 indicates.

**Table 1:** Inhibitory halos of the 100% OE and e dilutions against RC and C.

Dissolution 100%	No of repetitions	Inhibition halos (diameter in mm)			p
		Mean ± S.D	Variance	Median	
OE-RC	3	0.00 ± 0.00	310.08	0	0.038
e-RC	3	5.67 ± 4.51	20.33	6	
OE-C	2	0.00 ± 0.00	0.00	0	

Test of Kruskal*-Wallis, *p<0,05

The largest inhibition halo of the 75% dilutions was of the oily extract of *S. molle* against resistant *Candida albicans* with a value of (0.733 ± 0.461), however, the p value was greater than 0.05 (*p=0.177) indicating that there is no statistically significant dissimilarity as shown in Table 2.

**Table 2:** Inhibitory halos of the solutions with OE at 75% versus RC and C.

Disolution 75%	No of repetitions	Inhibition halos (diameter in mm)			p
		Mean ± S.D	Variance	Median	
OE-RC	3	0.73 ± 0.46	0.21	1	0.177
e-RC	3	0.67 ± 0.58	0.33	1	
OE-C	2	0.00 ± 0.00	0.00	0	

Test of Kruskal*-Wallis, *p>0,05

In the dilutions obtained at 50%, the highest value of the mean (2 ± 0) was presented in the oily extract of *S. molle* versus sensitive *C. albicans*, despite this it is statistically not significant since the p value was greater than 0.05 (*p=0.158) as detailed in Table 3.

**Table 3:** Inhibitory halos of the solutions with OE at 50% against CR and C.

Disolution 50%	No of repetitions	Inhibition halos (diameter in mm)			p
		Mean ± S.D	Variance	Median	
OE-RC	3	1.57 ± 1.72	2.96	1	0.158
e-RC	3	0.33 ± 0.58	0.33	0	
OE-C	2	2.00 ± 0.00	0.00	2	

Test of Kruskal*-Wallis, *p>0,05

The greatest inhibition halo was obtained with respect to the 25% solutions when using the oily extract of *S. molle* against sensitive *C. albicans* (1 ± 1.414) compared to the effect achieved with resistant *Candida albicans* (*p=0.702), as detailed in Table 4.

**Table 4:** Inhibitory halos of OE and e solutions at 25% against CR and C.

Disolution 25%	No. de repetitions	Inhibition halos (diameter in mm)			p
		Mean ± S.D	Variance	Median	
OE-CR	3	0.67 ± 0.58	0.33	1	0.702
e-CR	3	0.33 ± 0.58	0.33	0	
OE-C	2	1.00 ± 1.41	2.00	1	

Test of Kruskal*-Wallis, *p>0,05

**Figure 6:**
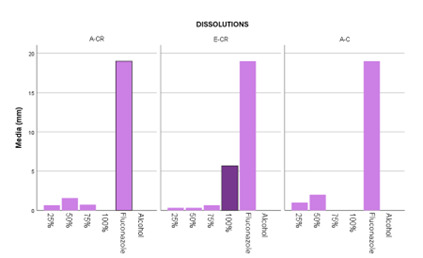
Comparison between extracts and concentrations

In this research, in spite of the values in millimeters obtained for the halos present in the different concentrations, it was found that there was an antifungal effect in the different percentages as can be seen in Figure 6, the most representative being the 100% ethanolic extract on resistant *Candida albicans*, with an average inhibition halo of 6 mm. This showed us that there was an inhibitory effect of the oily and ethanolic extract of *S. molle* in most of the solutions against resistant and sensitive *C. albicans*, that is, clinically it was significant, although statistically it shows the opposite in most of these concentrations.

## DISCUSSION

Currently, plant species have acquired increasing relevance in the field of phytotherapy due to their multiple therapeutic benefits and the low incidence of side effects associated with their use. Among the most outstanding applications are oily extracts and ethanolic extracts, which have demonstrated efficacy in the treatment of various conditions, particularly those related to the stomatognathic system. In particular, the ethanolic extract acts on the ergosterol pathway, thus causing fungal lysis. Within this framework, this research project aimed to quantify the inhibitory effect of the oily extract of *S. molle* against *Candida albicans*, based on previous scientific evidence documented by several authors. ^2^^,^^7^^,^^13^^,^^33^

Loyola et al. ^7^ carried out an in vitro study in which they used *Schinus molle* and Erythroxylum coca extracts, observing an antibacterial effect with their ethanolic extract at 50% and 75% on *S. mutans*, bacteria that are generally accompanied by *C. albicans* in carious lesions in patients with fixed orthodontics, which differs from this study because although minimal halos were observed at 50% and 75% against *Candida albicans*, statistically non-significant results were obtained with these concentrations.

Ferrerira et al. ^40^ carried out a study in 2023 of *Schinus weinmanniifolia* (Molle'i), a plant belonging to the same family as *S. molle* (Anacardiaceae), on the bacterial effect of *C. albicans*, obtaining that at concentrations of 25mg/ml they presented inhibitory effect, somewhat contrary to our values since we did not obtain inhibitory effect at concentrations of 25%.

Similar studies that have explored alternative treatments can be found in the literature, including the publication by Cadena-Viteri et al. ^2^ In this study, the antibacterial effect of *Schinus molle* on *Porphyromonas gingivalis*, a microorganism prevalent in people with periodontal disease, was evaluated. The results showed a highly sensitive inhibitory effect, reaching 100% efficacy up to 72 hours. These findings agree with the products resulting from this project, which determined that the ethanolic extract of *S. molle* at 100% concentration presented a greater inhibitory effect against resistant *Candida albicans*. ^2^

Likewise, Da Silva et al. ^32^ conducted a study in which it was reported that Cleome spinosa showed an inhibitory effect against *Candida albicans* yeasts when high concentrations of 6.25 and 12.5 mg/ml were used. These results are in quantitative agreement with the findings of this research project, in which a 100% inhibitory effect was achieved using ethanolic extract.

On the other hand, Tahtamouni ^41^ carried out an investigation on the ethanolic extract of *Schinus molle* obtained from different parts of the plant, evaluating its effect against various microorganisms. The results showed that ethanolic extracts obtained from the fruit and leaves presented a greater inhibitory effect compared to those from the branches and flowers. These qualitative findings coincide with the results of this study, since the ethanolic extract derived from the leaves of this plant species was used.

Kubizna et al. ^14^ conducted a systematic investigation that reported in the literature that curcumin, a bioactive compound extracted from turmeric, possesses outstanding anti-inflammatory, antioxidant, anticancer, antifungal and antimicrobial properties. Its main mechanism of action lies in altering the integrity of cell membrane proteins. In addition, curcumin has the ability to effectively absorb light, and when combined with blue light it acts as a potent photomodulator with an antifungal effect against *Candida albicans*. However, further studies are suggested, as a definitive protocol regarding extract concentration and light-curing time has not yet been established. In contrast, the present in vitro study was able to determine the specific percentage at which the ethanolic extract of *Schinus molle* could exert an antifungal effect.

Hsuan et al. ^13^ reported a systematic review of several plants with antifungal power on *Candida albicans*, being the most effective several plant species such as *G. xanthochymus*, *S. orientalis*, *Ex Hornem*, *M. alternifolia*, *C. nucifera*, among others. In which they determined that most of the plants with ethanolic extraction exhibited greater antifungal efficacy, however, it is consistent with the results obtained in this study seeing a statistically significant inhibitory effect only at a 100% concentration of ethanolic extract.

## CONCLUSIONS

The 100% ethanol extract of *C. albicans* resistant *Schinus molle* showed antifungal activity against resistant strains of *Candida albicans*, being classified as sensitive according to the Duraffourd scale, although with a lower efficacy compared to fluconazole, as shown by the smaller diameter of the inhibition halos. These results highlight the therapeutic potential of *S. molle*, although they also underline the need to optimize its formulation to increase its clinical effectiveness. In this context, phytotherapy represents an emerging therapeutic alternative of great interest, particularly in the field of dentistry, where antimicrobial resistance poses a growing challenge. Ecuador's vast plant biodiversity constitutes a valuable source of bioactive compounds with potential applications in the treatment of oral infections, which reinforces the importance of continuing to explore and scientifically validate these natural resources.

## Financing:

Self-financed
